# Urban malaria in sub-Saharan Africa: dynamic of the vectorial system and the entomological inoculation rate

**DOI:** 10.1186/s12936-021-03891-z

**Published:** 2021-09-08

**Authors:** P. Doumbe-Belisse, E. Kopya, C. S. Ngadjeu, N. Sonhafouo-Chiana, A. Talipouo, L. Djamouko-Djonkam, H. P. Awono-Ambene, C. S. Wondji, F. Njiokou, C. Antonio-Nkondjio

**Affiliations:** 1 Institut de Recherche de Yaoundé (IRY), Organisation de Coordination Pour la Lutte Contre les Endémies en Afrique Centrale (OCEAC), P.O. Box 288, Yaoundé, Cameroun; 2 Faculty of Sciences, University of Yaoundé I, P.O. Box 337, Yaoundé, Cameroon; 3 Faculty of Health Sciences, University of Buea, Cameroon, P.O. Box 63, Buea, Cameroon; 4 Faculty of Sciences, University of Dschang Cameroon, P.O. Box 67, Dschang, Cameroon; 5 Vector Group Liverpool School of Tropical Medicine Pembroke Place, Liverpool, L3 5QA UK

**Keywords:** Malaria, Urbanization, Sub-Saharan Africa, *Anopheles*, Entomological inoculation rate, Bionomic

## Abstract

Sub-Saharan Africa is registering one of the highest urban population growth across the world. It is estimated that over 75% of the population in this region will be living in urban settings by 2050. However, it is not known how this rapid urbanization will affect vector populations and disease transmission. The present study summarizes findings from studies conducted in urban settings between the 1970s and 2020 to assess the effects of urbanization on the entomological inoculation rate pattern and anopheline species distribution. Different online databases such as PubMed, ResearchGate, Google Scholar, Google were screened. A total of 90 publications were selected out of 1527. Besides, over 200 additional publications were consulted to collate information on anopheline breeding habitats and species distribution in urban settings. The study confirms high malaria transmission in rural compared to urban settings. The study also suggests that there had been an increase in malaria transmission in most cities after 2003, which could also be associated with an increase in sampling, resources and reporting. Species of the *Anopheles gambiae* complex were the predominant vectors in most urban settings. Anopheline larvae were reported to have adapted to different aquatic habitats. The study provides updated information on the distribution of the vector population and the dynamic of malaria transmission in urban settings. The study also highlights the need for implementing integrated control strategies in urban settings.

**Supplementary Information:**

The online version contains supplementary material available at 10.1186/s12936-021-03891-z.

## Background

Sub-Saharan Africa still bears the highest burden of malaria morbidity and mortality worldwide despite improvements in the diagnostic of the pathogens and large-scale deployment of vector control measures, such as Long-Lasting Insecticidal Nets (LLINs) and Indoor Residual Spraying (IRS) [1]. In 2019 over 229 million cases and 409,000 deaths were recorded across the world [1]. Although the whole sub-Saharan Africa region is exposed to malaria transmission risk, high heterogeneity in malaria transmission patterns exists on the continent, particularly between urban and rural settings [2–4]. It is considered that people living in rural settings are more exposed to malaria transmission risk compared to those living in urban settings [3, 5]. Studies conducted so far suggested higher densities and a greater diversity of malaria vector population sizes in rural compared to urban settings [6]. Many factors have been reported to affect malaria transmission intensity in urban settings including pollution, which can affect anopheline larval habitats and reduce their population size as well as impact mosquito life cycles and consequently their vectorial capacity. Urban dwellers may also have greater mosquito avoidance behaviour, including the use of repellents, screens on windows, insecticides spray and coils [2, 7–9]. During the last decade, sub-Saharan Africa registered an unprecedented growth of it urban population.

The urban population which was estimated at 491 million in 2015 is projected to grow to nearly 1.5 billion by 2050 [10]. However the rapid unplanned urbanization observed in many sub-Saharan Africa cities characterized by the colonization of lowland areas for house construction, the absence of drainage system for water, the presence of standing water collection everywhere due to the bad state of roads and poor housing are all considered to affect the distribution of vector population and malaria transmission pattern [11]. Now, many cities are reporting increase practice of urban agriculture in both the city centre and periphery; all these activities create favourable breeding habitats for mosquitoes [12–14]. The rapid unplanned urbanization appears as a potential risk factor promoting malaria and arboviral diseases transmission in urban settings [15, 16]. Studies conducted so far suggested higher densities and a greater diversity of malaria vectors in rural compared to urban settings [2, 6, 17]. Besides, it has been reported that the most efficient malaria vectors *Anopheles gambiae *sensu lato (*s.l*.) namely *Anopheles gambiae*, *Anopheles coluzzii*, *Anopheles arabiensis,* which had a strong preference for unpolluted water [13] now displays a great adaptation pattern to polluted waters in urban cities [18, 19] and breed in different human-made habitats including containers filled with water, swimming pools, tyre tracks, water tanks [20]. Housing construction sites or construction materials were also found to be productive habitats for malaria vectors [21, 22]. The situation of malaria in sub-Saharan African cities is further becoming complex with the recent invasion of the Asian malaria vectors *Anopheles stephensi* [23, 24]. Although the epidemiological consequences of such invasion is still not well understood, it is likely that the addition of new competent vectors in the urban environment may further negatively affect malaria control strategies in urban areas. Considering the potential public health impact that urban malaria could have and potential effects on the economic development of countries, there have been during the last decade a renewed interest with several studies assessing malaria transmission pattern and vector bionomic in urban settings across sub-Saharan Africa [11, 18, 21, 25–29]. However there have been so far not enough studies summarising findings from previous works in order to capture the general trend of malaria transmission, vector distribution and larvae preferred breeding habitats in urban settings. For instance, there have been fewer studies providing an overview of the general distribution pattern of main species in urban settings across Africa. The present study’s objective is to carry out a review of the existing literature on malaria transmission across sub-Saharan Africa in order to provide a better understanding of the evolution of vector populations and malaria transmission pattern.

## Methods

### Literature search

A search for studies on urban malaria in Africa was conducted to capture the general trend of malaria transmission using the following online databases PubMed, Research Gate, Google scholar and Google. Search terms included a combination of key words such as “EIR Anopheles”, “Urban malaria”, “malaria urban SSA”, “Africa mosquitoes”, “malaria transmission”, “Urbanization”, “cities”, “malaria *P. falciparum*”, “malaria epidemiology”, “urban population’, “malaria prevalence”.

### Literature selection process

**Fig. 1:**
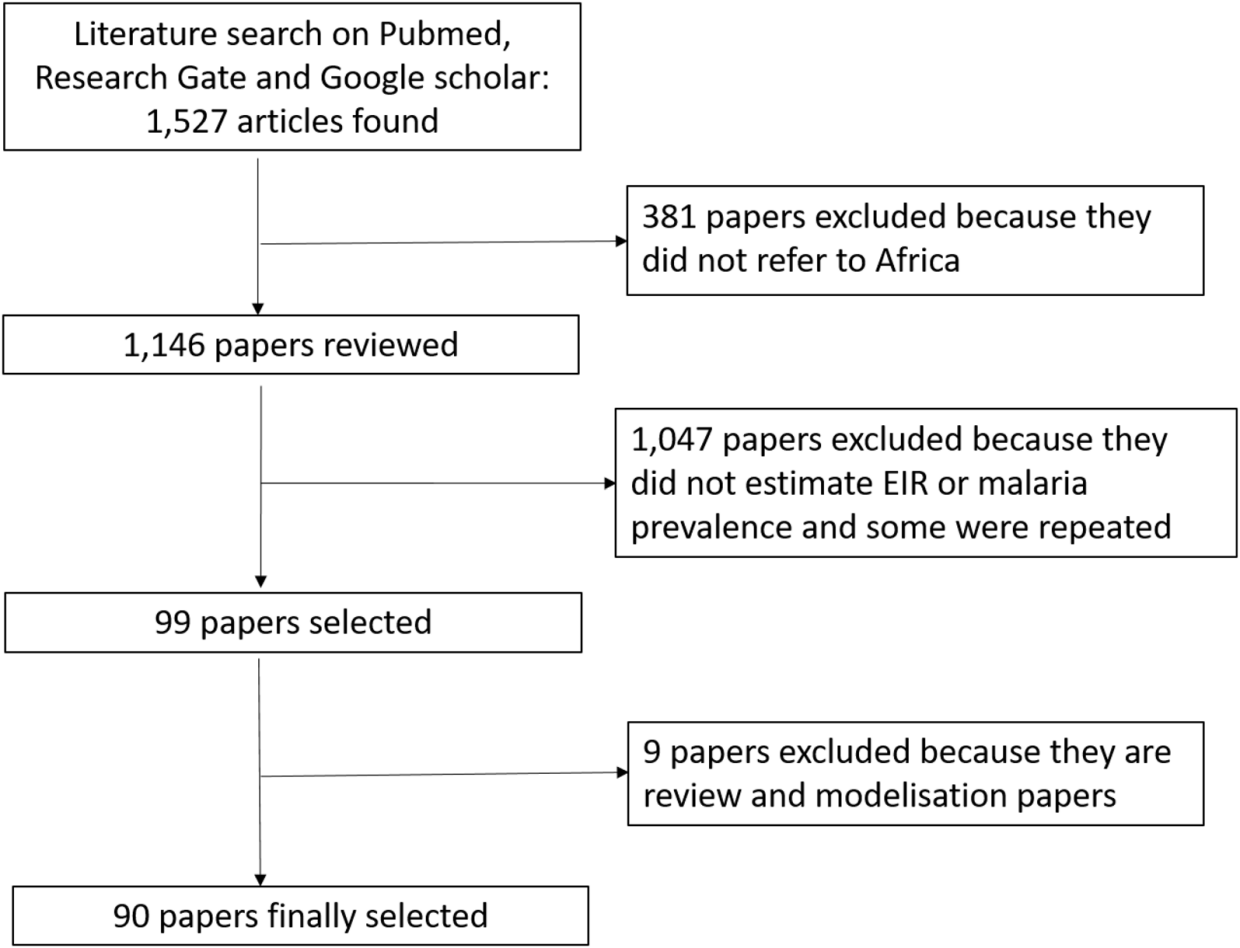
Flow chart of the literature selection process

The selection included papers published between the 1970s to 2020. Initial selection using the above combination of keywords yielded a total of 1527 scientific publications. All studies not conducted in Africa were discarded (n = 381). The remaining papers were then selected using the following criteria (i) description of malaria transmission or prevalence in urban and/or rural and/or periurban settings; (ii) studies conducted in sub-Saharan Africa. Papers which did not estimate the EIR or malaria prevalence or appearing several times in the selection were also excluded from the review. After applying this selection criteria, 1047 studies were discarded. Further reading of the abstract and the whole paper permitted to exclude an additional 9 papers living 90 for the study (Fig. 1).

Besides, an additional selection was performed to collate information on breeding habitats and anopheline species distribution in urban cities. Over 200 scientific publications were consulted for this purpose. Terms used to guide this search included the name of main cities in West, East and Central Africa followed by a combination of key words such as “malaria Anopheles”; “malaria Anopheles larvae”, “Anopheles urban breeding sites”, “Anopheles aquatic habitats”.

### Data analysis

Available information retrieved from each selected publication were registered in a Microsoft Excel spreadsheet for data analysis. This included authors names, the year of publication, country, study site, types of settings, sampling method, study type, malaria transmission indices (entomological inoculation rate (EIR), human biting rate (HBR)), vectors involved in the transmission, abundance of vectors, study period, malaria prevalence, and parasites (Additional file 1).

EIRs estimates were not always available in selected papers in an adequate format for analysis. The following steps were taken in order to adjust data presentation. (i) When many EIRs were estimated for the same site (EIRs for districts within a city), the average EIR from the area was estimated and used for analysis; (ii) when the EIR value was presented for two different periods in the same site, the highest value was considered; (iii) when indoor and outdoor EIRs were reported, the EIRs were summed to have the total EIR from the area; (iv) when EIRs were presented as daily or monthly or seasonal EIRs, the annual EIR was estimated.

The Spearman correlation coefficient was used to assess the correlation between EIRs and biting rates. The Kruskal–Wallis and Mann–Whitney tests were used to compare EIRs averages between urban, periurban and rural settings. The EIR was also compared between periods before 2003 and after 2003 because after 2003 studies conducted were using more molecular tools for mosquito processing (PCR, ELISA) than before. Analyses were performed using R software version 3.4.0. and GraphPad Prism 7.

### Study design

**Fig. 2:**
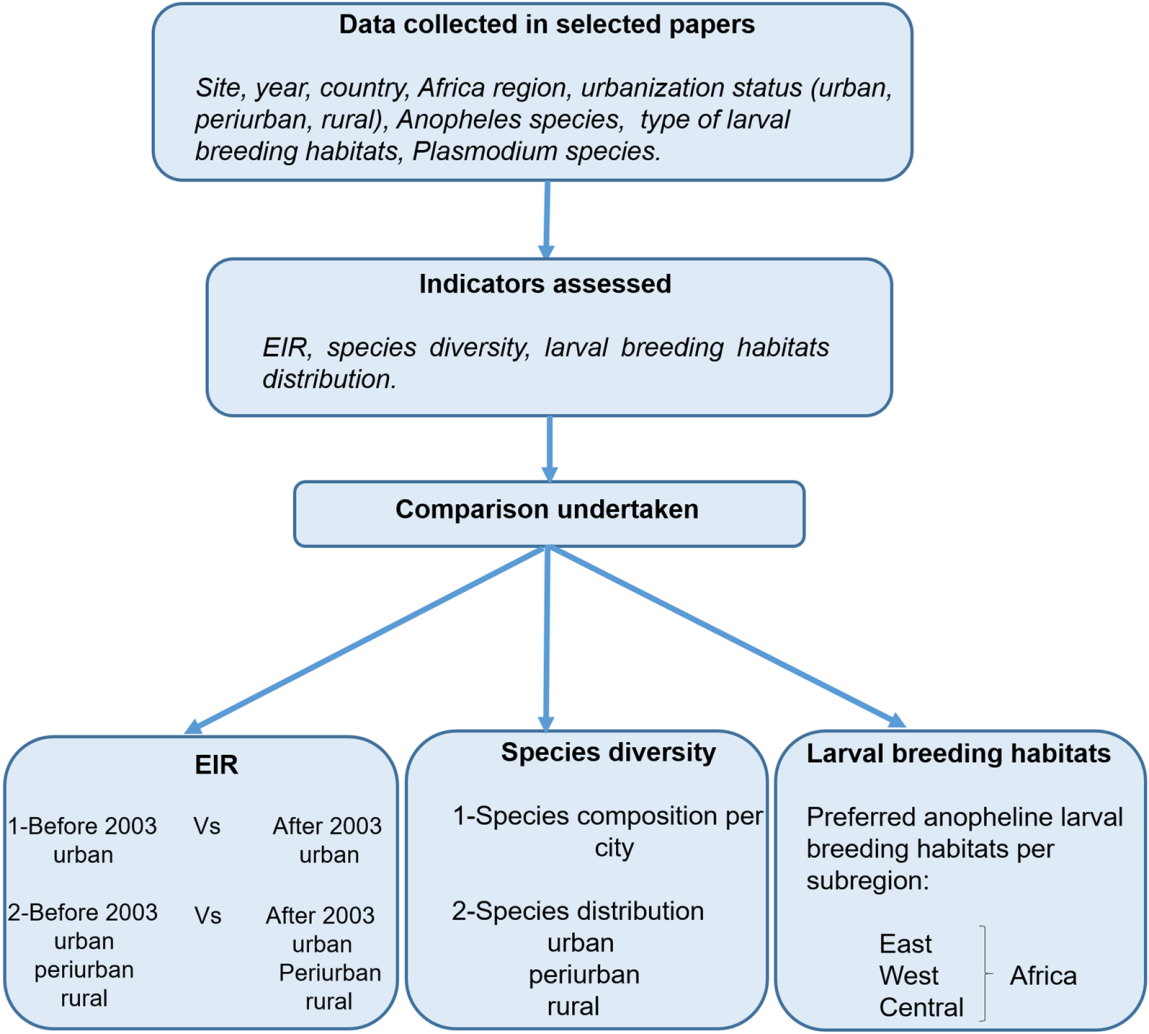
Study design

A flowchart representing the study design show data collected in selected papers, indicators assessed and comparisons performed in the review (Fig. 2).

## Results

### Literature review of EIR estimates in rural, periurban and urban settings

A total of 90 studies conducted in 136 sites in 23 countries were consulted for the present review (Table 1). Data presented derive from studies in 88 rural sites, 18 periurban sites and 31 urban sites.

**Table 1:** EIRs estimates in different studies conducted across sub-Saharan Africa between 1977 and 2020

Authors	Year	Country	Locality	EIR		
				Rural	Periurban	Urban
Abraham et al. [78]	2017	Ethiopia	Sille	63.6		
Akogbeto et al. [79]	2000	Benin	Cotonou	12	47	29
Adja et al. [80]	2011	Côte d'ivoire	Gbatta, Kpehiri	298.8; 478.8		
Akono et al. [30]	2015	Cameroon	Akonolinga, Yaoundé		813.95	552.61
Akono et al. [81]	2015	Cameroon	Logbessou		47.28	
Antonio-Nkondjio et al. [82]	2002	Cameroon	Simbock	368		
Antonio-Nkondjio et al. [14]	2012	Cameroon	Douala			31
Amawulu et al. [83]	2016	Nigeria	Bayelsa			80.5
Amek et al. [84]	2012	Kenya	Nyanza	25.6		
Amvongo-Adjia et al. [85]	2018	Cameroon	Tiko, Manfe, Santchou			8.4;16.8;26.88
Appawu et al. [86]	2004	Ghana	Kassena Nankana	1218		
Beier et al. [45]	1990	Kenya	Kisian, Saradidi	299; 237		
Bockarie et al. [87]	1994	Sierra Leone	Bo	21–36		
Cano et al. [31]	2004	Equatorial Guinea	Bioko			814.27
Cano et al. [88]	2006	Equatorial Guinea	Yengue	298.8		
Carnevale et al. [89]	1985	Congo	Brazzaville	80–850		
Carnevale et al. [90]	1992	Cameroon	Mbebe	182		
Coene [91]	1993	RD Congo	Kinshasa	455		30
Degefa et al. [92]	2015	Ethiopia	Jimma	0–4781.5		
Diallo et al. [93]	1998	Senegal	Dakar			0
Diallo et al. [94]	2000	Senegal	Dakar			0
Daygena et al. [95]	2017	Ethiopia	Gato	103.2		
Elissa et al. [96]	1999	Gabon	Franceville	365	81	
Epopa et al. [97]	2019	Burkina Faso	Bana, Pala, Souroukoudingan	393.47; 199.65; 151.84		
Getachew et al. [98]	2019	Ethiopia	Ghibe			13.8
Lwetoijera et al. [99]	2014	Tanzania	Kilombero	392.31		
Djamouko-Djonkam et al. [37]	2020	Cameroon	Yaoundé		106.83	9.78
Dossou-yovo et al. [100]	1995	Ivory Coast	Bouake	230		
Dossou-yovo et al. [101]	1994	Ivory Coast	Bouake		126; 88	
Doumbe-Belisse et al. [11]	2018	Cameroon	Yaoundé			0–92
Drakeley et al. [102]	2003	Tanzania	Ifakara		30.7	
Fontenille et al. [103]	1992	Madagascar	St Marie Island	100		
Fontenille et al. [104]	1997	Senegal	Dielmo	159		
Fontenille et al. [105]	1997	Senegal	Ndiop	31		
Fouque et al. [106]	2010	French Guinea	Loca, Twenke	10; 5		
Govoetchan et al. [107]	2014	Benin	Sonsoro, Gansosso	130.75		6.45
Hakizimana et al. [108]	2018	Rwanda	Karambi, Mashesha, Kicukiro	1–329.8		107.5
Himeidan et al. [109]	2011	Sudan	Koka, Um Salala	109.5; 3.65		
Karch et al. [110]	1992	Congo	Kinshasa	620	66	3
Kasasa et al. [111]	2013	Ghana	Navrongo			1.132–157
Kerah-Hinzoumbé [112]	2009	Chad	Goulmoun	311		
Kibret et al. [113]	2014	Ethiopia	Ziway	0.25–27.3		
Klinkenberg et al. [13]	2008	Ghana	Accra			6.6–19.2
Krafsur et al. [114]	1977	Western Ethiopia	Gambela	97		11
Lemasson et al. [115]	1997	Senegal	Barkedji	114		
Lindsay et al. [116]	1990	Gambia	Banjul			1.3
Lochouarn et al. [117]	1993	Burkina Faso	Bobo-Dioulasso			2
Gadiaga et al. [118]	2011	Senegal	Dakar			17.6
Githeko et al. [119]	1993	Kenya	Ahero	91–416		
Machault et al. [28]	2009	Senegal	Dakar			0–16.8
Mala et al. [120]	2011	Kenya	Kamarimar, Tirion	1.44; 1.61		
Manga et al. [121]	1992	Cameroon	Yaoundé			3; 13
Massebo et al. [122]	2013	Ethiopia	Chano	0–73.2		
Mbogo et al. [123]	2003	Kenya	Malindi	0–120		
Mbogo et al. [124]	1993	Kenya	Kilifi	8		1.5
Mbogo et al. [125]	1995	Kenya	Kilifi	0–59.6		
Mourou et al. [39]	2012	Gabon	Libreville			33.9
Mourou et al. [41]	2010	Gabon	Libreville, Port-Gentil			3.45;66.45
Mutuku et al. [126]	2011	Kenya	Kidomaya, Jego	5.16		
Muturi et al. [127]	2008	Kenya	Kiamachiri, Mbuijeru, Murinduko	4.06; 2.55; 2.50		
Mwangangi et al. [128]	2013	Kenya	Kimudia, Kiwalwa, Mwarusa, Njoro	31.95; 123.92; 59.78; 45.06		
Mwanziva et al. [129]	2011	Tanzania	Gichameda	0.51		
Ndenga et al. [130]	2006	Kenya	Iguhu, Kombewa,Marani,Mbale	16.6;31.1;0.4;1.1		
Njan Nloga et al. [131]	1993	Cameroon	Ebogo	355		
Okello et al. [132]	2006	Uganda	Jinja, Arua, Apac, Tororo, Mubende, Kyenjojo, Kanungu	397; 1586; 562; 4; 7; 6	6	
Okwa et al. [133]	2009	Nigeria	Bungudu-Gusau, Badagry, Onitsha, Bonny	23.31	74.1;32.1; 34.5	
Olayemi et al. [134]	2011	Nigeria	Ilorin and Minna			0.83
Overgaard et al. [135]	2012	Equatorial Guinea	Bioko			163–840
Owusu-Agyei et al. [136]	2009	Ghana	Kintampo	269		
Richard et al. [137]	1988	Congo	Mayombe		80; 397	
Robert et al. [89]	1985	Burkina Faso	Bobo-Dioulasso	50; 60; 55; 133		
Robert et al. [138]	1986	Burkina Faso	Bobo-Dioulasso		5	0.1;0.5
Robert et al. [139]	1993	Cameroon	Edea			4; 30
Robert et al. [140]	1998	Senegal	Niakhar	9; 12;26		
Rossi et al. [141]	1986	Burkina Faso	Ouagadougou	92; 82; 430	10; 23	7;0;0
Salako et al. [50]	2018	Benin	Alibori, Donga	285.48		49.8
Shiff et al. [142]	1995	Tanzania	Coastal Tanzania	94–703		
Shililu et al. [143]	2003	Eritrea	Anseb, Debub, Gash-Barka, Nothern Red Sea	3.45; 15.95; 66.45; 0		
Smith et al. [144]	1993	Tanzania	Kilombero	329		
Tabue et al. [145]	2017	Cameroon	Garoua, mayo Oulo, Pitoa	71.54	33.9	3.45
Tanga et al. [146]	2010	Cameroon	Likoko	460.1		
Tchouassi et al. [147]	2012	Ghana	Kpone-on-sea	62.1		
Tchuinkam et al. [51]	2010	Cameroon	Djuttitsa, Dschang, Santchou	0; 90.5		62.8
Thompson et al. [148]	1997	Mozambique	Maputo		20	0
Trape and Zoulani [149]	1987	Congo	Brazzaville		101	0.3
Trape et al. [150]	1992	Senegal	Pikine, Dakar		0.4	0.01
Vercruysse [151]	1981	Senegal	Pikine, Dakar			43
Vercruysse [152]	1985	Senegal	North Senegal	1; 6.5		
Yadouléton et al. [12]	2010	Benin	Cotonou, Parakou, Porto Novo			102.2; 54.73;83.95
Zogo et al. [153]	2019	Côte d'ivoire	Korhogo	2.46		

EIR, infected bites per person per year

### Dynamic of the EIR between rural, periurban and urban settings

**Fig. 3:**
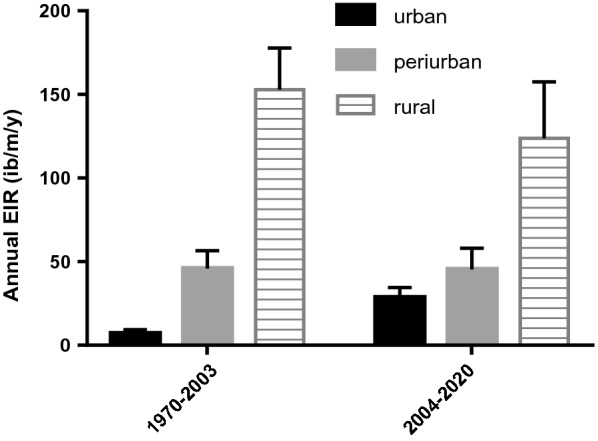
Evolution of malaria transmission from 2003 onwards. Errors bars represents 95% confidence interval

A high heterogeneity in EIR estimates was recorded between urban, periurban and rural settings. The average EIR was 144.05 infective bites per person per year (ib/p/yr) 95% CI [141.9–146.22] in rural area, 45.70 ib/p/yr 95% CI [42.86–48.7] in periurban area and 32.73 ib/p/yr 95% CI [31.13–34.38] in urban sites. A significant difference was recorded when comparing the EIR between rural, periurban and urban areas (P ˂ 0.05). When comparing EIR estimates of the urban centre between the period before 2003 (1977–2003) and the period after 2003 (2004–2020) it appears a significant increase in malaria transmission estimates in urban centres since 2003 (P = 0.0001), no such increase was recorded for periurban and rural settings (Fig. 3).

### Evolution of EIR in main sub-Saharan Africa cities

**Fig. 4:**
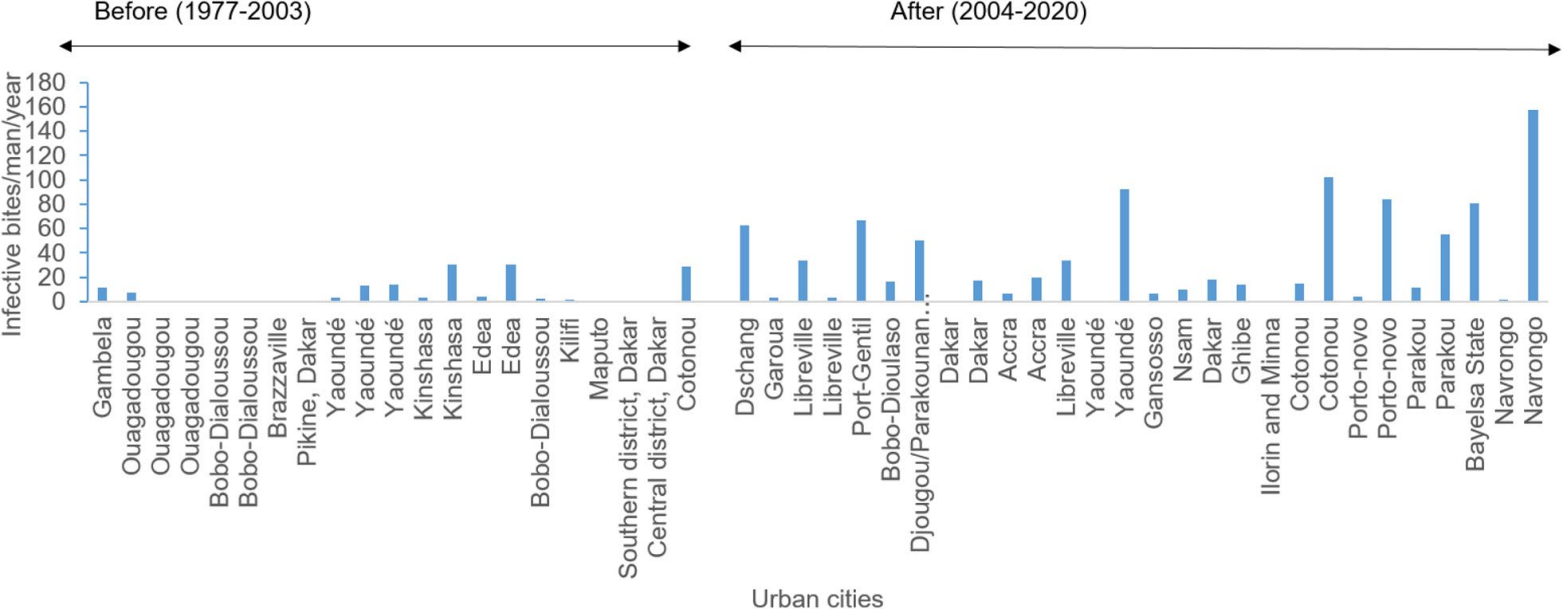
Variation of Entomological inoculation rate in urban cities

When comparing the EIR estimates in urban centres between 1977 and 2020, it appears that before 2003 there were many cities reporting very low or no transmission of malaria from mosquitoes to man whereas between 2004 and 2020 almost all studies indicated evidence of malaria transmission (Fig. 4). Average EIR values varying annually from 30 to 100 ib/p/yr were recorded in most urban settings (Fig. 4). Extreme values of EIR above 500 ib/p/yr have also been reported in Yaoundé [30] and Bioko [31], but these values were not included in the present analysis. *Plasmodium falciparum* was the main parasite detected in most cases.

### Larval sites distribution in urban area

*Anopheles* larvae were frequently found in man-made water habitats, such as drains, puddles, market gardens, urban agricultural sites, pools drains and tyre tracks. *Anopheles* larvae were also reported in natural breeding sites such as swamps, streams or rivers bed although they are less common and scattered in urban areas. The number of studies highlighting the specific breeding sites of anopheline in urban areas both natural and artificial is presented in Table 2.

**Table 2:** Type of breeding habitats found with anopheline larvae in urban settings across sub-Saharan Africa

	Type of breeding site	West Africa	References	Central Africa	References	East Africa	References
Artificial	Urban farms	10	[12, 13, 18, 21, 26, 28, 154–157]			4	[27, 158–160]
	Tyre tracks	4	[18, 28, 161, 162]	1	[19]	6	[22, 158, 160, 163–165]
	Drains/gutter	4	[154, 159, 162, 166]	4	[19, 167–169]	5	[170, 158, 159, 164, 165]
	Swimming pool	1	[21]			4	[22, 27, 159, 171]
	Pools	4	[161, 162, 166, 172]	1	[168]	5	[160, 164, 165, 173, 174]
	Polluted water	3	[13, 18, 155]	1	[19]	1	[175]
	Pipes	1	[13]			2	[176, 177]
	Dam	1	[155]	1	[178]		
	Brick holes	1	[155]				
	Domestic containers	2	[162, 166]	2	[19, 168]	3	[163, 165, 179]
	Footsprint			2	[19, 167]	2	[163, 165]
	Ditches/pits	1	[154]	1	[169]	4	[163–165, 174]
	Rice paddies			1	[180]	2	[163, 165]
	Puddles	1	[154]	2	[19, 169]	4	[159, 164, 173]
	Holes					1	[164]
	Canoes			2	[168, 169]		
Total		33		18		43	
Natural	Swamps	3	[28, 154, 155]	1	[19]	4	[159, 160, 163, 164]
	Streams/rivers/lagoon	6	[21, 154, 156, 161, 32, 181]	1	[182]	3	[159, 164, 173]
	Ponds	1	[161]	2	[178, 183]	1	[159]
	Well	1	[13]	1	[168]		
	Ground water/springs					2	[27, 158]
	Tree holes			2	[166, 168]	1	[184]
	Clay soil	1	[21]			1	[27]
	Flood plain/ravine	1	[155]			1	[171]
Total		13		7		13	

### Diversity of the *Anopheline fauna* in urban cities

**Fig. 5:**
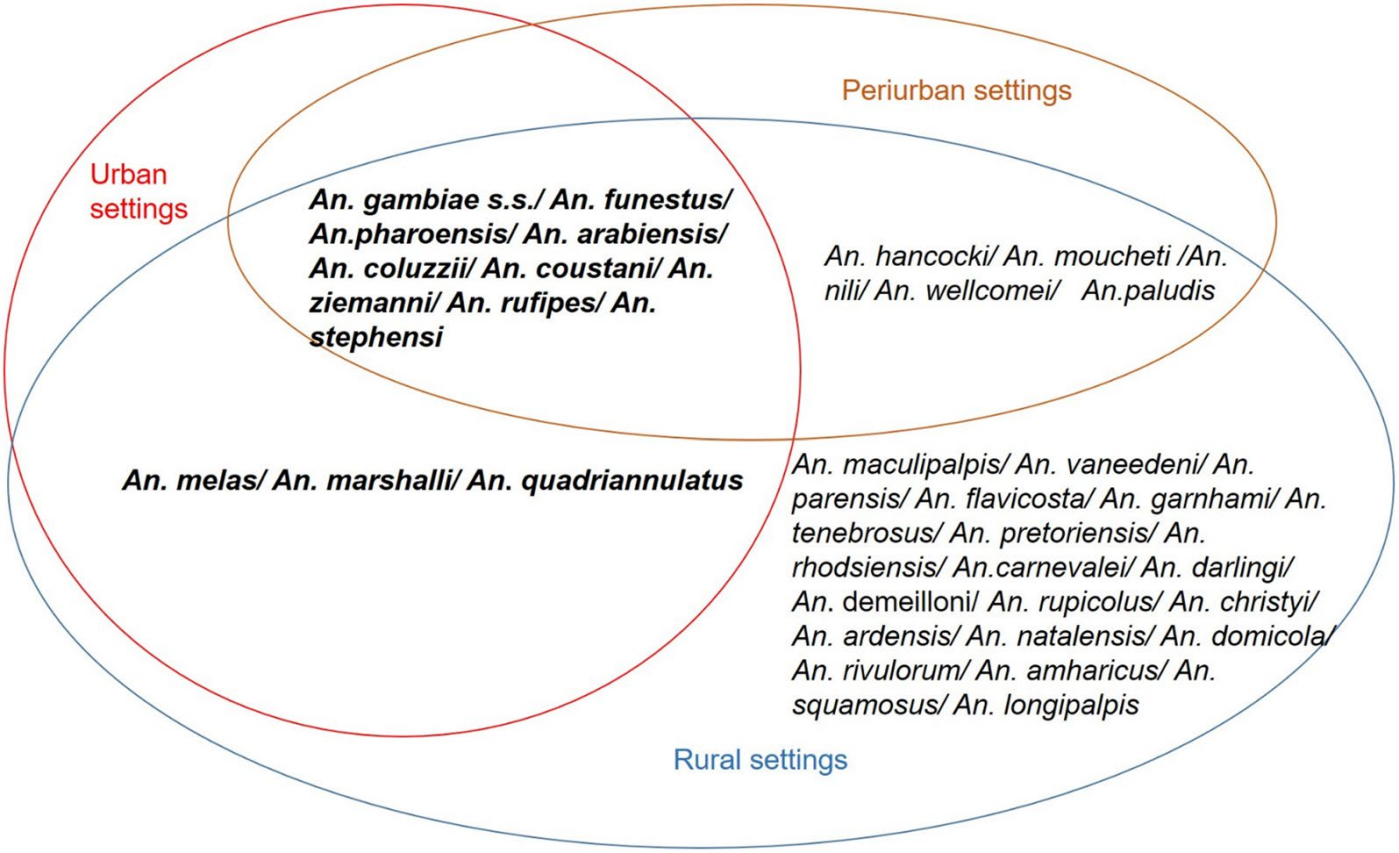
Distribution of anopheline species between rural, periurban and urban settings

A total of 12 anopheline species were reported in studies conducted in urban cities. Species of *An. gambiae* complex, including *Anopheles gambiae *sensu stricto (*s.s*.), *Anopheles coluzzii* and *Anopheles arabiensis* were the most common. Additional species present in urban settings included *Anopheles melas*, *Anopheles funestus s.s*. and *Anopheles stephensi*. Other anopheline species such as *Anopheles coustani*, *Anopheles ziemanni*, *Anopheles marshallii,* and *Anopheles rufipes* were reported, but in very low densities (Table 3). Great diversity and higher densities of species in rural areas compared to periurban and urban centres were recorded (Fig. 5). For instance, in the city of Yaoundé, it was common to find fewer than four species at the city centre whereas this number could rise up to ten species in the nearby rural settings.

**Table 3:** Composition of anopheline species recorded in main cities across Africa

Africa subregion	Country	Cities	Main species (> 90% total)	Others species < 10%	References
Central Africa	Cameroon	Garoua	*An. gambiae* s.s	*An. rufipes/An. pharoensis/An. funestus/An. paludis*	[145, 180, 52]
		Yaoundé	*An. gambiae* s.s./*An. coluzzii/An. funestus*	*An. nili/An. marshalli/An. ziemanni/An.moucheti*	[11, 19, 30, 37, 121, 185, 59, 186]
		Douala	*An. coluzzi*	*An. gambiae* s.s.*/An. ziemanni*	[14, 167, 168]
	Gabon	Libreville	*An. gambiae* s.s		[39, 41]
		Port-Gentil	*An. melas/An. gambiae* s.s		[41]
		Franceville	*An. funestus/An. gambiae* s.s		[96]
	Equatorial Guinea	Bioko	*An. funestus/An. gambiae* s.s	*An. melas*	[31, 135, 187–191]
	Tchad	N’Djamena	*An. gambiae* s.s.*/An. arabiensis/An. coluzzii*		[192–194]
	Angola	Lobito	*An. coluzzii/An. gambiae* s.s		[195, 196]
		Luanda	*An. gambiae* s.s		[197]
	Congo	Brazzaville	*An. gambiae* s.s	*An. moucheti*	[149]
	Democratic Republic of Congo	Lodja/Kapolowe	*An. gambiae* s.s		[198]
		Kinshasa	*An. gambiae* s.s.*/An. funestus/An. paludis*	*An. moucheti/An. nili*	[110, 199–201]
		Kibali	*An. gambiae* s.s.*/An. funestus*		[202]
	Central Africa Republic	Bangui	*An. coluzzii/An. gambiae* s.s*./ An. funestus* s.s		[203–206]
	Benin	Cotonou	*An. gambiae* s.l	*An. pharoensis/An. ziemanni/An. funestus*	[12]
West Africa		Porto Novo	*An. gambiae* s.l	*An. pharoensis/An. ziemanni/An. funestus*	[12]
	Côte d’ivoire	Yamoussoukro	*An. gambiae* s.l	*An. funestus*	[207]
		Abidjan	*An. gambiae* s.s		[207]
		Bouaké	*An. gambiae* s.s		[208, 209]
	Gambia	Bakau	*An. arabiensis/An. coluzzii*	*An. gambiae s.s./An. gambiae* s.s. and *An. coluzzii hybrids*	[210]
	Senegal	Dakar	*An. gambiae* s.l.*/An. arabiensis*	*An. pharoensis/An. ziemanni* *An. melas/An. gambiae* s.s	[28, 118, 211]
		Kedougou	*An. coustani/An. funestus*	*An. domicola/An. flavicosta/An. gambiae* s.l./*An. hanckocki*/*An*. *nili*/*An. rufipes*/*An. wellcomei*	[212]
	Guinea	Conakry	*An. coluzzii*/*An. gambiae* s.s		[54, 213, 214]
		Siguiri	*An. gambiae* s.s./*An. funestus*	*An. arabiensis*	[215]
	Guinea-Bissau	Bissau	*An. gambiae* s.s./*An. coluzzii/An. arabiensis*	*An. melas/An. pharoensis*	[216–220]
	Mauritania	Nouakchott	*An. gambiae* s.s./ *An*. *arabiensis*/*An. pharoensis*		[221, 222]
	Burkina Faso	Bobo-Dioulaso	*An. arabiensis*	*An. coluzzii/An. gambiae* s.s	[223, 223–225]
		Ouagadougou	*An. gambiae* s.l./*An. coluzzii*/*An. arabiensis*		[154, 226, 227]
	Cabo-Verde	Praia	*An. arabiensis*		[228, 229]
	Liberia	Montserrado	*An. gambiae* s.s	*An. coluzzii*	[230, 231]
		Monrovia	*An. gambiae* s.s./*An coluzzii*	*An. funestus*	[232]
	Nigeria	Ilorin and Minna	*An. gambiae* s.l		[134]
		Lagos	*An. gambiae* s.s.*/An. arabiensis*	*An. rivulorum* /*An. funestus*	[233–236]
		Bayelsa	*An. gambiae* s.s		[83]
	Mali	Bamako	*An. coluzzii/An. gambiae*	*An. arabiensis*	[237–239]
	Ghana	Accra	*An. gambiae* s.s.*/An. coluzzii*	*An. funestus/An. coustani*	[13, 170, 240]
	Niger	Niamey	*An. gambiae* s.s.*/An. arabiensis*	*An. funestus/An. rufipes/ An. pharoensis/ An. ziemanni*	[40]
		Tessaoua	*An. coluzzii*		[194]
	Togo	Lomé	*An. gambiae* s.s.*/An. coluzzii*		[241]
	Ethiopia	Kebri Dehar	*An. stephensi*		[23, 242]
		Arjo-Didessa	*An. arabiensis*	*An. amharicus/An. coustani /An. pharoensis/An. squamosus /An. funestus*	[159, 243]
	Djibouti	Djibouti	*An. arabiensis/An. stephensi*		[244, 245]
	Sudan	Khartoum	*An. arabiensis*		[246–250]
	Tanzania	Dar es Salaam	*An. arabiensis*	*An. funestus/An. gambiae* s.s*.*/*An. coustani*	[55, 251]
		Morogoro	*An. arabiensis*	*An. gambiae* s.s./*An. coustani /An. quadrianulatus*	[162, 252]
East Africa	Kenya	Nairobi	*An. arabiensis/An. funestus*		[253, 254]
		Kisumu	*An. funestus*	*An. rivulorum/ An. leesoni, An. parensis/ An. longipalpis/An. vaneedeni*	[255]
		Kilifi	*An. funestus*	*An. rivulorum/An. leesoni, An. parensis, An. longipalpis/An. vaneedeni*	[255, 256]
	Rwanda	Kigali	*An. arabiensis*	*An. funestus/An. ziemanni/An. coustani /An.moucheti An. gambiae* s.s	[108, 257]
	Burundi	Karuzi	*An. gambiae* s.s	*An. demeilloni*/*An. arabiensis/An. funestus*	[258]
	Uganda	Tororo	*An. arabiensis*	*An. gambiae* s.s	[259]

## Discussion

The present study is an update of previous reviews on urban malaria in sub-Saharan Africa [2–4, 29, 32, 33], it provides new data on malaria transmission pattern and anopheline species distribution. Urbanization is increasingly blamed of influencing the epidemiology and evolution of vector-borne diseases in sub-Saharan Africa. More than half of the world’s population now lives in towns or cities and it is projected that this number could rise to 75% by 2050 [34]. From the review it appears that, the Entomological Inoculation Rate (EIR) is highly heterogeneous in cities across the continent [18, 35, 36]. In many cities centre, malaria transmission is low or absent while others register high EIR estimates [11, 37]. The difference between cities could derive from the scale of urban development, population size and the magnitude of unplanned urbanization. Unplanned urbanization characterized by the colonization of lowland areas for habitat construction, poor drainage system in urban settings, the development of slums and spontaneous habitats and the practice of agriculture in the city centre was reported to deeply influence malaria transmission intensity [15, 38]. Although EIR estimates were always higher in rural and peri-urban settings compared to urban centres [2], it also appeared that, because of increase poverty in urban settings there are an increasing number of people exposed to malaria transmission risk. In the city of Libreville for instance, a high transmission rate was recorded in the city centre characterized by poor housing, high population density, low socio-economic level and inadequate management of waste, compared to the periphery where the population had a high socio-economic level and good management infrastructure [39]. In the city of Yaoundé, where slum-like conditions are common across the city, malaria transmission was highly prevalent in both the city centre and the periphery [11]. The close association between malaria and the economic status of the household has been highlighted in different studies across the continent [4]. Additional factors including river overflowing, city landscape and seasonal variations were also found to influence the intensity and pattern of malaria transmission [14, 40]. Important differences were noted when comparing malaria transmission intensity in the city centre before and after 2003. The comparison of the two periods suggested an increase in malaria transmission intensity in urban settings across sub-Saharan Africa after 2003 [11, 13, 31, 39, 41]. Transmission estimates surpassing 50 infected bites/person/year were frequently reported in cities across Africa supporting the existence of high parasite reservoirs in urban settings including migrants coming from highly endemic rural settings or population moving from urban to rural settings which could be infecting mosquito populations [30, 37]. It is also possible that the introduction of new techniques for the detection of *Plasmodium* infections in mosquitoes, such as ELISA and PCR techniques which were not used before could have increased the EIR estimates [42–44]. These highly sensitive techniques were reported to overestimate the true infection rate after salivary gland dissection by 1.1 to 1.9 folds [45–48]. The use of new molecular techniques or genomic advances could be vital for malaria control and elimination in Africa and there is a need to promote the use of new techniques to improve malaria vector control and surveillance in Africa [49].

The study also indicated high diversity of the vectorial system in different cities [12, 50–53]. Yet members of *An. gambiae* complex were largely predominant in most urban settings [11, 14, 54, 55]. This is in conformity with these species capacities to adapt to anthropogenic and/or environmental changes and to feed exclusively on humans [56]. The preferential breeding habitats of species of the *An. gambiae* complex are temporary water collections exposed to sunlight. However, these species were reported to also breed in different types of habitats, including drains, septic tanks, artificial containers, standing water collection full of organic matters in urban settings [2, 15]. Moreover, it appears from the study that species composition could vary significantly between cities [51]. The following observation, highlights the influence of different factors genetic and tolerance level shaping the adaptation capacity of species in different environments [57, 58]. In the city of Yaoundé, the predominance of *An. coluzzii* over *An. gambiae* was attributed to the high tolerance of the species to organic pollutants, such as ammonia [59]. In coastal cities along the Atlantic Ocean, such as Libreville and Malabo, *An. gambiae* was found to be highly predominant whereas it was less abundant in Douala where *An. coluzzii* was the predominant species [60]. Explaining species distribution relying only on species specific data could be more complex as highlighted in a recent meta-analysis [61] and deserve further investigation. Urban agriculture coupled with uncontrolled disposal of containers to collect rainwater is creating an increasing number of favourable aquatic breeding habitats for Anopheles in urban cities. It has been reported that some *Anopheles* species are now adapting to this new environment, as described for *An. stephensi*, which breeds in man-made water containers, such as household water storage containers and garden reservoirs [24, 62]. The invasion of Africa by new species, such as *An. stephensi,* which is now found in many countries across East Africa such as Djibouti, Somalia, Sudan, and Ethiopia, could pose a great challenge for malaria elimination in Africa particularly in urban settings [1, 23, 63–65]. The invasion of Djibouti by *Anopheles stephensi* in 2012 was associated with a 30-fold increase in malaria cases, from 1684 in 2012 to 49,402 in 2019 [1]. *Anopheles stephensi* was also reported to display high resistance to pyrethroids, carbamates and organophosphates [64]. The species bites outdoors and displays a highly opportunistic behaviour feeding on both human and animals, a behaviour which could affect the efficiency of current control measures [64].

Some cities exhibited a high species diversity with three to six species commonly reported whereas low species diversity was recorded in others. This heterogeneity between cities could derive from difference in vegetation, altitude, urbanization level and seasons [66–70]. The presence of forest fringes as observed in the close neighbourhood of some cities [51] could increase the number of potential breeding sites exploited by mosquitoes and explain the diversity. Mosquitoes found in the urban environment are also exposed to a high selection pressure induce by the use of insecticide-treated nets, pollution, deforestation, anthropogenic changes and environmental changes which could reduce the diversity and distribution of species [15]. Indeed high intensity insecticide resistance affecting almost all insecticide families was reported in *An. gambiae s.l.* populations from most urban settings [12, 19, 71–73]. The rapid expansion of multiresistance pattern was reported to reduce bed nets efficacy in different epidemiological settings [74–76]. In urban settings where vector populations display resistance to insecticide, and outdoor feeding behaviour [11], the addition of targeted interventions such as larval control in hotspot areas could be keys for effective reduction of malaria transmission.

*Plasmodium falciparum* was the predominant malaria parasites recorded in almost all urban settings. This parasite is also the dominant species in rural settings [77]. Other species commonly found included *Plasmodium malariae* and *Plasmodium ovale* [53]. It is likely that the diversity of *Plasmodium* species in urban settings could be on the rise due to the intensification of travels between different regions of the globe. The exploration of factors favouring mosquito nuisance and malaria transmission in urban settings clearly shows the influence of urban expansion resulting from rapid population growth outpacing infrastructure development and highlight the need for further action by municipalities and public works services in the construction of drains or sewage systems to reduce breeding opportunities for mosquitoes [13].

## Conclusion

The current review provides an update of the situation of malaria in urban settings in sub-Saharan Africa during the last decades. Although the risk of malaria transmission remains low in urban compared to rural settings, urban malaria is likely to increase as unplanned urbanization continues. Unplanned urbanization led to a proliferation of suitable breeding habitats for malaria vectors and thus increases the risk of exposition to mosquito bites and malaria transmission. To stop this trend in the disease burden, concerted actions need to be taken quickly at different levels to improve the management of malaria cases and control of vector populations. The development of integrated control approaches could be paramount for the effective control of vector-borne diseases in urban settings.

## Supplementary Information

**Additional file 1.** Database of the entomological inoculation rate estimates reported in the 90 scientific publications selected for the review.

## Data Availability

Not applicable.

## Abbreviations

EIRs: Entomological Inoculation Rate
PCR: Polymerase Chain Reaction
ELISA: Enzyme-Linked Immunosorbent Assay
